# Natural History and Management of Familial Paraganglioma Syndrome Type 1: Long-Term Data from a Large Family

**DOI:** 10.3390/jcm9020588

**Published:** 2020-02-21

**Authors:** Giulia Puliani, Franz Sesti, Tiziana Feola, Nicola Di Leo, Giorgia Polti, Monica Verrico, Roberta Modica, Annamaria Colao, Andrea Lenzi, Andrea M. Isidori, Vito Cantisani, Elisa Giannetta, Antongiulio Faggiano

**Affiliations:** 1 Department of Experimental Medicine, Sapienza University of Rome, 00161 Rome, Italy; giulia.puliani@uniroma1.it (G.P.); franz.sesti@uniroma1.it (F.S.); tiziana.feola@uniroma1.it (T.F.); andrea.lenzi@uniroma1.it (A.L.); andrea.isidori@uniroma1.it (A.M.I.); elisa.giannetta@uniroma1.it (E.G.); 2 Department of Radiological, Pathological and Oncological Sciences, Sapienza University of Rome, 00161 Rome, Italy; nicola.dileo130287@gmail.com (N.D.L.); giorgia.polti@gmail.com (G.P.); monica.verrico@virgilio.it (M.V.); vito.cantisani@uniroma1.it (V.C.); 3 Department of Clinical Medicine and Surgery, University of Naples Federico II, 80131 Naples, Italy; robertamodica@libero.it (R.M.); colao@unina.it (A.C.)

**Keywords:** familial paraganglioma syndrome type 1, SDHD, paraganglioma, neuroendocrine neoplasm

## Abstract

Head and neck paragangliomas are the most common clinical features of familial paraganglioma syndrome type 1 caused by succinate dehydrogenase complex subunit D (SDHD) mutation. The clinical management of this syndrome is still unclear. In this study we propose a diagnostic algorithm for SDHD mutation carriers based on our family case series and literature review. After genetic diagnosis, first evaluation should include biochemical examination and whole-body imaging. In case of lesion detection, nuclear medicine examination is required for staging and tumor characterization. The study summarizes the diagnostic accuracy of different functional imaging techniques in SDHD mutation carriers. 18F-3,4-dihydroxyphenylalanine (18F-DOPA) positron emission tomography (PET)-computed tomography (CT) is considered the gold standard. If it is not available, 123I-Metaiodobenzylguanidine (MIBG) could be used also for predicting response to radiometabolic therapy. 18F-fluoro-2-deoxy-D-glucose (18F-FDG) PET-CT has a prognostic role since high uptake identifies more aggressive cases. Finally, 68Ga-peptides PET-CT is a promising diagnostic technique, demonstrating the best diagnostic accuracy in our and in other published case series, even if this finding still needs to be confirmed in larger studies. Periodic follow-up should consist of annual biochemical and ultrasonographic screening and biannual magnetic resonance examination to identify biochemical silent tumors early.

## 1. Introduction

Familial paraganglioma syndrome type 1 (FPGL1) is a rare autosomal dominant disorder associated to succinate dehydrogenase complex subunit D (SDHD) germline mutations. Clinical features of FPGL1 are head and neck paragangliomas (PGLs) and neuroendocrine neoplasms arising from parasympathetic paraganglia in 85% of cases, while more rarely, thoraco-abdominal PGLs from sympathetic paraganglia (in 20–25% of cases) and pheochromocytomas (in 10–25%), with a low malignancy rate (4%) [1]. Considering patients affected by pheochromocytomas, mutation in SDHD is found in 2,3% of cases [2]. Nowadays, the causal relationship between SDHD mutations and familial paraganglioma syndrome type 1 has been demonstrated [3]. More than 130 intragenic mutations, single or multiple exon deletions and intragenic duplications, have been identified in SDHD genes. The SDHD gene encodes the D subunit of the SDH enzyme, which is a part of mitochondrial complex II and plays a critical role in the Krebs cycle and respiratory chain electron transport [4]. Modifications of this pathway increase concentration of intermediate substrates and alter cell metabolism, leading to activation of the angiogenic pathway, DNA hypermethylation, and alteration of the tumor microenvironment [5].

This syndrome is rare, with an estimated prevalence of 1–9/1,000,000 and the management remains unclear. Although genetic and physiopathological aspects of SDHD mutations have been deeply studied, current pheochromocytomas and PGLs clinical guidelines [6] do not focus specifically on the clinical management of FPGL1 patients.

The aim of this study is to describe the natural history and management of a case series deriving from a large FPGL1 family.

## 2. Patients and Methods

The present case series included multiple members of a family with SDHD-positive PGLs referred to outpatient clinics of the specialized multidisciplinary neuroendocrine team NeuroEndocrine Tumor TAsk foRcE (NETTARE Unit), Policlinico Umberto I, Sapienza University Hospital of Rome. For all the patients, the SDHD mutation had been confirmed using a DNA mutation analysis thanks to the collaboration with the Azienda Ospedaliero-Universitaria di Careggi (Florence, Italy) and by AMES group of Naples, using direct DNA sequencing with the Sanger method on an ABI-PRISM 350^®^ and subsequent data analysis with sequencing analysis version 6 (Applied Biosystems^TM^). Clinical characteristics including sex, age at clinical diagnosis, age at genetic familial screening, age at last follow-up, location, size and number of SDHD-related tumors, laboratory results, conventional and functional imaging data, histopathological examination, local and systemic treatments, and length of follow-up were collected.

A written informed consent for publication was signed from all patients. All data were collected and used in conformity with the European General Data Protection Regulation 2016/678. Ethical Committee of Sapienza University of Rome do not require a specific approval process for case series studies.

## 3. Results

### 3.1. Family Case Series

**Figure 1:**
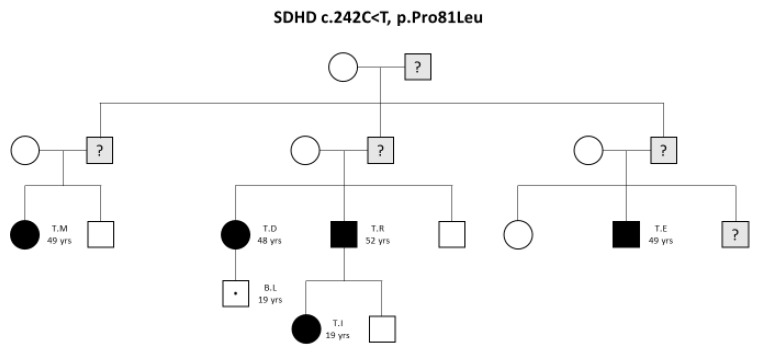
Family tree. Abbreviation: SDHD, succinate dehydrogenase complex subunit D.

In a FPGL1 family with SDHD mutation, c.242C < T, p.Pro81Leu, 5 subjects (3 females and 2 males), were found to be affected by one or more PGLs (Figure 1).

### 3.2. Clinical Presentation and Diagnosis

Of the 5 affected family members, 3 patients referred the first clinical symptoms (swelling in lateral neck region) at a median age of 40 years (range 19–51), receiving the diagnosis of PGLs through the morphological imaging (7 neck PGLs and 1 mediastinal PGL) and were subsequently genetically confirmed. The other 2 subjects received the diagnosis of FPGL1 during the familial genetic screening, at the age of 14 and 47 years respectively, before clinical evidences. In these two subjects who had no evidence of disease at diagnosis, left neck PGLs occurred after 5 and 4 years, respectively. At the time of the clinical diagnosis, all patients were asymptomatic (symptoms related to neuroendocrine secretion or mass) and the biochemical screening for neuroendocrine markers was negative (chromogranin A -CgA, neuron specific enolase, urinary metanephrines). Until now, 13 PGLs has been globally detected in the five patients, 11 of them being identified at the morphological imaging and 2 at only functional imaging. Clinical characteristics of the five patients are reported in Table 1.

### 3.3. Imaging Work up

All the patients performed morphological imaging with color-doppler ultrasound (US) and computed tomography (CT), and/or magnetic resonance (MR) angiography of the neck. Suspected lesions at US were always confirmed with CT or MR imaging. All patients underwent a functional imaging investigation with whole-body positron emission tomography (PET)-CT scan, as summarized in Table 2, which globally identified 13 lesions, two more than morphological imaging.

**Figure 2:**
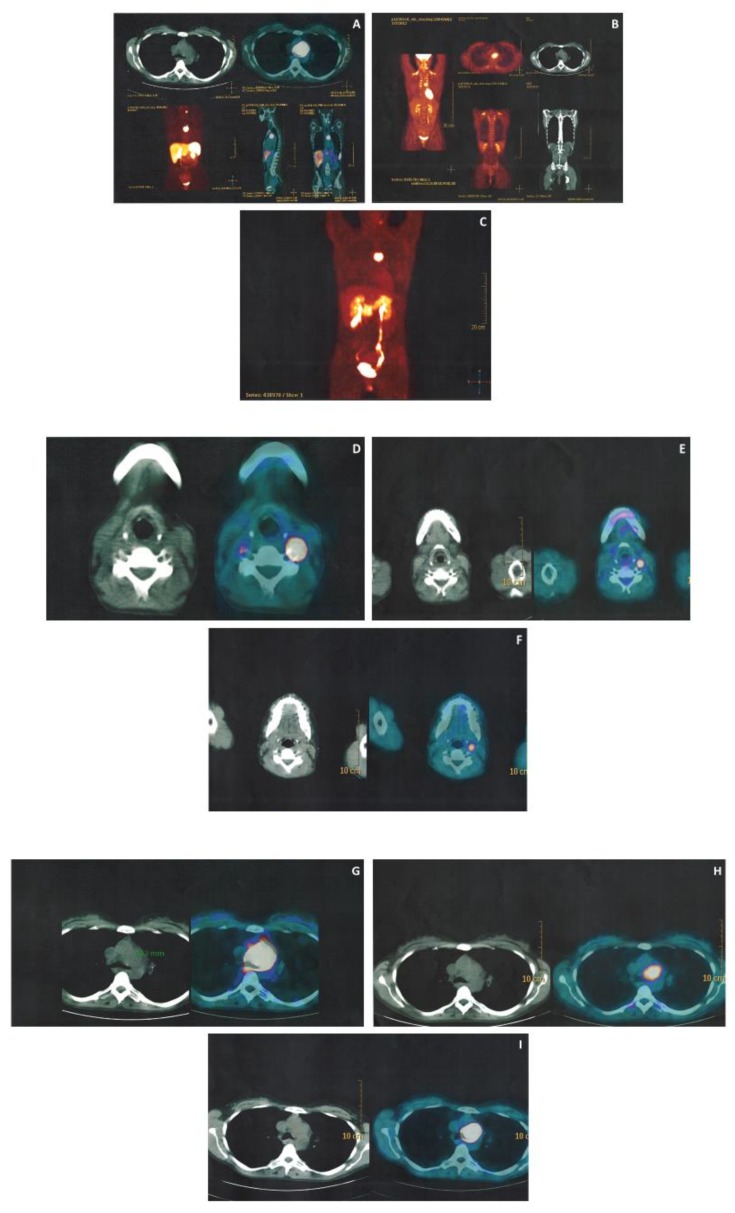
PET-CT comparison: (**A**,**D**,**G**): 68GaDOTATOC; (**B**,**E**,**H**): 18F-FDG; (**C**,**F**,**I**): 18F-DOPA. Abbreviations: PET = positron emission tomography; CT = computed tomography; DOTATOC = 1,4,7,10-tetraazacyclododecane-1,4,7,10-tetraacetic acid tyr3- Octreotide; FDG = fluoro-2-deoxy-D-glucose; DOPA = 3,4-dihydroxyphenylalanine.

Four patients (cases 1–4) were studied with 68Gallium-1,4,7,10-tetraazacyclododecane-1,4,7,10-tetraacetic acid tyr3- Octreotide (68Ga-DOTATOC) PET-CT, detecting 9 lesions. Two of which are newly diagnosed, without evidence from the morphological imaging, found early identification of PGLs based on the focal uptake in areas expected to develop PGLs while excluding false positive results. Three patients (cases 1, 3, 4) were studied with 18F-fluoro-2-deoxy-D-glucose (18F-FDG) PET-CT, detecting 5 of the 6 lesions, previously identified with morphological imaging (mean standardized uptake value, SUV, max was 9.2). Two patients (cases 1, 5.) were studied with 18F-3,4-dihydroxyphenylalanine (18F-DOPA) PET-CT, detecting 3 of the 4 lesions, previously identified by the morphological imaging. In one patient (case 1) performing 68Ga-DOTATOC, 18F-FDG and 18F-DOPA PET-CT, only the 68Ga-DOTATOC PET-CT detected all the three lesions, identified with morphological imaging, while 18F-FDG and 18F-DOPA PET-CT detected only 2 of them (Figure 2). Two patients (cases 3, 4) performed both 68Ga-DOTATOC and 18F-FDG PET-CT, which were able to detect all the known lesions.

### 3.4. Treatment and Follow-up

**Figure 3:**
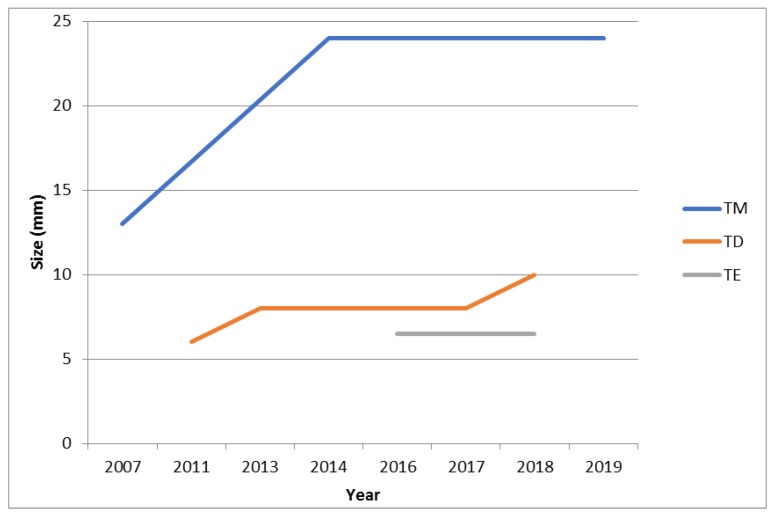
PGLs size change during follow-up. Abbreviation: PGLs = paragangliomas.

8 PGLs were surgically excised and histologically confirmed. In the patient with mediastinal PGL, surgery was followed by adjuvant radiotherapy and medical therapy with somatostatin analogs (SSA), octreotide long-acting release treatment (30 mg/28 days), because of the positivity of resection margins. All the excised PGLs were not relapsed. Three PGLs did not undergo surgery due to their small size: 1 was stable at 7-year follow-up (from 6 to 10 mm) in the patient who received SSA treatment after surgery and radiotherapy of the mediastinal PGL; 1 was slowly progressive at a 7-year follow-up (increasing by about 1 cm, from 13 to 24 mm), 1 was stable at a 2-year follow-up (from 6 to 6.5 mm) (Figure 3). Two PGLs were diagnosed with functional imaging at the last follow-up. The median follow-up was 44 months (range 15–156 months) performed by outpatient visits, biochemical and instrumental evaluations. All patients had benign PGLs without evidence of metastases and no other SDHD-linked neoplasms were detected.

Patients’ characteristics and PGLs natural history and management are summarized in Table 1. Functional imaging findings are summarized in Table 2.

## 4. Discussion

### 4.1. Natural History

FPGL1 is caused by the mutation in the SDHD gene and it is characterized by the development of PGLs, in the most of cases localized in the head and neck region. PGLs normally present after the age of 30 [7]. However, in a series of 177 pediatric patients (17 SDHD) from the European American Pheochromocytoma Paraganglioma Registry, the average age at SDHD-related PGLs diagnosis was 14 years (95%, CI 12–15), and one case had the diagnosis at 5 years old [8]. Mean life expectancy of SDHD mutation carriers was not calculated, because no patients died during the follow-up. A second and a third tumor was developed by 59% and 24% of SDHD patients, respectively [8]. Accordingly, in case of adult onset, SDHD-related lesions are usually multifocal [9]. In our series, four of five patients (80%) had multiple lesions.

In FPGL1, malignant rate, defined as the presence of PGL cells in nonchromaffin organs [9], is low. A recent metanalysis calculated a pooled incidence of malignant PGLs of 8% (95% CI, 2–26%) and a pooled risk in prevalence studies of 4% (95% CI, 2–7%) for SDHD-mutation carriers [10]. Considering only SDHD-related PGLs (without asymptomatic carriers) the pooled prevalence of malignancy was 3% (95% CI, 1–10%) [10].

More rarely, SDHD mutation carriers can develop other kinds of tumor. The most frequent is renal cancer tumor, with a lifetime risk of 8% [11], followed by gastrointestinal stromal tumors (GISTs). In an unselected clinical record, SDH-deficient GISTs account for 5% of all gastric GISTs [12] and presented peculiar characteristics: the predominant cell type is epithelioid (not spindled), GISTs are often multifocal, with frequent lymph nodes dissemination, and younger age at diagnosis [13]. Only in a few cases there was an association between pituitary adenomas and PGLs [14]. In the current series, all but one PGLs developed in the head and neck (one in mediastinum) and no other tumor type was identified, despite the extensive morphological and functional imaging work-up.

### 4.2. Current Diagnostic Work-up

#### 4.2.1. The Role of Serum and Urinary Markers

Clinical management of SDHD mutation carriers is challenging. Current Endocrine Society guidelines [6] suggest annual biochemical surveillance for pheochromocytomas and PGLs, assessing plasma free metanephrines or urinary fractionated metanephrines.

Biochemical examination can include plasma free metanephrines (consisting of metanephrines, MN and normetanephrines, NMN), methoxytyramine (3-MT), or 24 h urinary fractionated metanephrines [15]. Data on the diagnostic accuracy of 3-MT are contrasting: even if same initial data suggested that the dosage of plasma 3-MT can increased the detection rate of pheochromocytomas and sympathetic PGLs, especially in SDHB and SDHD mutation carriers [16], a recent prospective study by Rao et al. [17] on 1963 screened subjects showed that 3-MT could increase false positive results. In particular in head and neck PGLs, adding 3-MT causes a small decrease in specificity and a slight increase in sensitivity. This data is due to only 11/38 patients with head and neck PGLs who showed increases only in plasma concentrations of 3-MT. However, none of the patients that were positive for 3-MT were SDHD mutation carriers [17].

Serum CgA is considered optional [15]. A recent study on the role of CgA for surveillance of SDHB- and SDHD-related PGLs, performed on 62 patients (52 SDHB and 10 SDHD), demonstrated a clinical utility of CgA in SDHB patients (sensitivity 67% and specificity 79%) but not in SDHD group (sensitivity 22% and specificity 0%) [18].

Our case series is in accordance with these data: in all patients with or without PGLs, CgA was negative.

#### 4.2.2. The Role of Magnetic Resonance Imaging

Although computed tomography (CT) is a commonly used diagnostic technique, recently published recommendations stated that it should be reserved for circumstances where biochemical abnormalities are detected and MR is contraindicated, due to its ionizing radiation burden and exposure to iodine contrast medium [15].

In case of familiar PGLs, periodic morphological imaging with MR is recommended considering the possibility of biochemical silent tumors [19,20,21]. However, no specific indication on timing is provided [6].

Recently, American recommendations on surveillance in childhood with hereditary pheochromocytomas and PGLs have been published [15], in which a specific surveillance protocol is recommended for SDH mutation carriers (irrespective of mutation type). In these guidelines, an annual biochemical screening and a biannual morphological screening with whole-body MR is recommended. Other reviews propose the same diagnostic algorithm [22,23].

Even if MR, when applicable, is the method of choice for morphological examination [6,15], there is no consensus on MR protocol. Considering the possibility of abdominal PGLs or pheochromocytoma also in FPGL1, some studies have evaluated the diagnostic accuracy and applicability of whole-body MR. In a study by Jasperson et al. [24], the whole-body MR protocol consists of axial and coronal ultra-fast T2 weighted sequence (“HASTE” technique: half-Fourier acquisition single-shot turbo spin echo) and demonstrated a sensitivity of 87.5% and a specificity of 94.7% in detecting lesions. The authors concluded that this whole-body MR protocol is accurate and time saving compared to single classical MR protocols. Coherently, Daniel et al., in a study on 47 patients with SDHx mutations (mainly SDHB) confirmed that whole-body MR is effective for lesion detection in this group of patients. In this study, MR protocol included also the axial T1 spin echo in-out phase sequence [25]. A recent review by Muth et al. inserted whole-body MR every 2 years in the diagnostic work-up of SDHD mutation carriers [23]. In our case series, neck MR failed to detect lesions only in two cases, which were identified with functional imaging (with a sensibility of 84.6% and specificity of 100%). A reasonable work-up could be to perform neck and mediastinum MR every two years and whole-body MR every 4–5 years. Alternatively, the whole-body MR could be proposed only in case of suspected abdominal tumors. This would avoid a heavy and maybe unhelpful imaging workup in subjects who are not expected to develop tumors outside the head or neck and mediastinum.

#### 4.2.3. The Role of Ultrasonography

Available guidelines do not recommend the use of US for the screening of neck PGLs in SDHD mutation carriers, even if US is the first line diagnostic procedure for various neck lesions. PGLs at US appear as clearly demarcated solid masses with a heterogeneous hypoechoic structure [26]; color-doppler examination shows hypervascularity with a low-resistance flow pattern [27]. In our cases, US was diagnostic in 11/13 lesions (84.6%), with the same detection rate of neck MR.

So far, there are not so many studies to clearly delineate the possible role of contrast enhanced ultrasonography (CEUS) in the diagnosis and follow-up of neck PGLs, Rübenthaler et al. [28] have reported the use of this technique in the assessment of a percutaneous embolization procedure of a neck PGL. These lesions normally show a strong and diffuse contrast enhancement, so the capability to effectively demonstrate the extent of devascularization immediately after the treatment can be very useful to monitor therapy success without using ionizing radiation.

Since silent neck PGLs are the most prevalent type of lesions in SDHD mutation carriers [7], neck US and CEUS could be part of radiological follow-up, also considering cost, feasibility, accessibility and patient agreement.

With regard to pheochromocytomas, the use of CEUS in the diagnosis of adrenal masses was reported to be below 70% [29]. A characteristic hypervascularity may be demonstrated along with a central core of lack of contrast uptake due to necrosis; a vascularized thrombus within the adrenal vein may also be demonstrated in case of aggressive tumors [30].

#### 4.2.4. Functional Imaging: “Back to the Future”

For the last few years, nuclear medicine has been assuming increasing relevance in the diagnosis of PGLs, especially after the publication of comparison studies on diagnostic accuracy rate of various techniques. 123I-Metaiodobenzylguanidine (MIBG) scintigraphy has been studied for more than 30 years and its accuracy has been established in various studies, including a prospective multicentric study [31], in which this technique demonstrated a sensitivity and specificity of 82% in the detection of PGLs and pheochromocytomas, leading it to become the nuclear imaging gold standard [32]. Nevertheless, considering only SDHx mutation carriers, the accuracy of morphological examinations (neck MR and CT scan of other regions) seemed better than scintigraphy (123I-MIBG and 111In-pentetreotide somatostatin receptor scintigraphy -SRS-) in all sites apart from thoracic lesions in which SRS demonstrated the best sensitivity [33]. Certainly, the spread of PET has changed this view. In extra-adrenal PGLs, 18F-DOPA PET-CT was superior to SRS in localizing small lesions [34] and superior to 18F-FDG in localizing SDHx-related head and neck PGLs [35]. Considering hereditary PGLs, 18F-DOPA PET-CT is a sensitive and specific imaging modality for the detection and staging of pheochromocytomas and PGLs and was able to identify PGLs in SDHD mutation carrier in 8/8 cases [36]. In all patients with PGLs, 18F-DOPA PET-CT is recommended for the detection of additional PGLs and for excluding metastatic spread [32]. Other studies reported that 18F-DOPA might be falsely negative in PGLs, especially those related to mutations in SDHD, and this lower uptake of 18F-DOPA by PGLs seems to be dependent on amino acid transport system L isoforms 3 and 4, which are less expressed in SDHD-mutated patients [37].

In recent years, 68Ga-DOTA-octreotate (DOTATATE) PET-CT was reported to show greater accuracy than morphological imaging [38] and other functional imaging, especially in SDHB-related PGLs, in which somatostatin analogues receptor type 2A and 3 are more strongly expressed [39]. However, data are available on a small number of patients [32]. Janssen et al., in a study on 20 patients with head and neck PGLs, reported higher accuracy of 68Ga-DOTATATE PET-CT than 18F-DOPA PET-CT, 18F-FDG, and 18F-fluorodopamine (18F-FDA) PET-CT, as well as classical morphological imaging (CT/MR) [40]. This finding was confirmed also on 23 patients with metastatic and sporadic disease [41]. Chang et al., in a study on 23 SDHx patients (1 SDHD), reported similar per lesion diagnostic performance of 68Ga-DOTATATE PET-CT and 18F-FDG PET-CT, but a significantly greater lesion-to background contrast in the former. Moreover, the combination of high FDG but low DOTATATE uptake can be a potential indicator of aggressive disease [42]. Finally, in the evaluation of 68Ga-peptides PET-CT accuracy, it is important to considerer the possibility of false positive results due to: (a) physiological uptake of pancreatic uncinate process and spleen, (b) uptake of non-tumor skeletal diseases (osteoarthritis, fractures, vertebral hemangioma), (c) inflammatory diseases and meningiomas [43]. In our cases, 68Ga-DOTATOC PET-CT had the best diagnostic rate, being able to detect 13/13 lesions (100%).

### 4.3. Our Novel Insight for A Precision Diagnostic Algorithm

**Figure 4:**
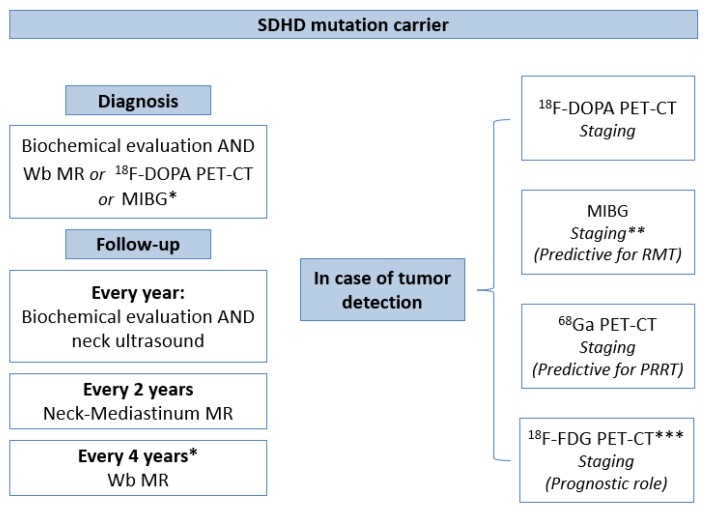
Proposed diagnostic algorithm; * Or in case of suspected symptoms of extra head/neck/mediastinum tumors; ** If DOPA not available; *** To define biological aggressiveness and metastatic potential. Abbreviations: Wb = whole body; MR = magnetic resonance; DOPA = 3,4-dihydroxyphenylalanine; PET = positron emission tomography; CT = computed tomography; MIBG = metaiodobenzylguanidine; RMT = radioactive microsphere therapy; PRRT = peptide receptor radionuclide therapy; FDG = fluoro-2-deoxy-D-glucose.

Based on the available evidence in literature, confirmed by our current experience, we propose a specific diagnostic algorithm for SDHD mutation carrier, as summarized in Figure 4.

Genetic diagnosis, biochemical evaluation, and MR examination are recommended. Biochemical evaluation should include serum MN and NMN or 24 h urinary fractionated metanephrines. Data on the usefulness of 3-MT are not enough strong for recommending the use of this examination in all SDHD mutation carriers, so the decision of what biochemical evaluation, plasmatic or urinary, is most appropriate and depends also on the available exams in each center. If no lesion is detected after the achievement of a genetic diagnosis, we recommend an annual screening with neck US and biochemical assessment, biannual neck-mediastinum MR examination, and a whole-body MR every 4 years. Considering that the most common lesions in FPGL1 are head and neck PGLs, neck MR could be an alternative imaging instead of whole-body MR, but not enough evidence is currently available.

After PGLs detection we recommend functional imaging, if not already performed, in order to evaluate possible metastatic spread or concomitant primary lesions and to guide a therapeutic decision. 18F-DOPA PET-CT is the most studied functional imaging, but it could be falsely negative in patients with SDHD mutations. Preliminary data seems to demonstrate a better accuracy of 68Ga-peptides PET-CT when compared to 18F-DOPA PET-CT in SDHD patients. Moreover, positivity on 68Ga-peptides PET-CT is needed for eventual somatostatin analogues or radionuclide treatment. In this view, we recommend 68Ga-peptides PET-CT. 123I-MIBG could be considered if the two other procedures are not available. 18F-FDG PET-CT could detect aggressive lesions and 123I-MIBG could identify candidates for radionuclide therapy.

### 4.4. Therapy

Surgical complete tumor excision is considered the treatment of choice in case of functioning non-metastatic PGLs [8]; in silent PGLs with low risk of malignancy, as in SDHD-mutated PGLs, all risks and benefits should be considered due to the possible complications of surgical treatment, for example cranial nerves lesions in neck PGLs [44]. In metastatic PGLs, 131I-MIBG is the most studied treatment, while radiolabeled somatostatin analogs have been used in small retrospective and prospective studies [9,45]. Chemotherapy (cyclophosphamide, vincristine, and dacarbazine) is indicated in case of rapidly progressive metastatic disease [46]. More recently, the antiangiogenic drug sunitinib, an inhibitor of multiple tyrosine kinase receptors, has been used in metastatic PGLs and 8/17 patients had partial response or stable disease [47]. Therapy with somatostatin analogues (octreotide and lanreotide), the mainstream of well differentiated neuroendocrine tumors treatment, in PGLs is understudied. In a case series of 4 patients, two of them with SDHD mutation, octreotide treatment was able to stabilize tumor size in one patient with SDHD mutation (50%) [48]. In our case series, 3 morphologically-detected lesions did not undergo surgical intervention. Considering the two patients with a longer follow-up (7 years), the patient treated with octreotide was substantially stable, while the untreated patient was slowly progressive.

## 5. Conclusions

The best clinical management of SDHD-related PGLs is not clearly defined due to the rarity of the disease, the lifelong necessity of follow-up, which contraindicated invasive imaging techniques, and the unreliability of biochemical biomarkers, considering the large proportion of silent PGLs. In this study, we tried to summarize the available evidence in the literature and our experience coming from this large family, in order to propose a diagnostic algorithm. A limitation of the study is the low number of patients involved. In SDHD mutation carriers, we suggest MR examination after genetic testing; periodic follow-up should include an annual biochemical and US screening and biannual neck-mediastinum MR examination. In case of lesion detection, nuclear medicine examinations are necessary for excluding other PGLs or metastases and for predicting responses to radionuclide therapy in metastatic or inoperable cases. 18F-DOPA PET-CT is actually the gold standard. However, 68Ga-peptides PET-CT is a promising diagnostic technique and could represent the biological base of somatostatin analogues therapy in SDHD-mutated PGLs, even if further studies are needed to confirm their clinical application.

## Figures and Tables

**Table 1:** Clinical characteristics. Abbreviations: yrs = years; dx = diagnosis; FU = follow-up; NA = not available; M = metanephines; NM=normetanephrines; CT = computed tomography; MR = magnetic resonance; PGL = paraganglioma; NSE = neuron specific enolase; CgA = chromogranin A; HPF = high-power field; OCT = octreotide.

Patient	Demographic Parameters: (1) Sex (2) Age at Clinical dx (yrs) (3) Age at Genetic dx (yrs) (4) Age at Last FU (yrs)	FU from Clinical Diagnosis (Months)	Tumor Lesions: (1) Number (2) Site	Size at Diagnosis (mm)	Basal Morphological Imaging	Urinary Fractionated Metanephrines	Histology	Surgery	Medical Therapy	Follow-up
**Case 1 (T.D.)**	(1) Female (2) 40 (3) 41 (4) 48	91	(1) 3 (2) a: left carotid bifurcation b: right carotid bifurcation c: mediastinum	a: 18 b: 6 c: 38	- echography - CT angiography - MR	M: 111 µg/24 h (50–340), N: 312 µg/24 (90–445)	a: PGL NSE+, synaptophysin +, CgA+, S100+; b:- c: PGL, synaptophysin +, CgA+, Ki67 < 1%, R1	a: yes, 2013 b: no c: yes, 2013 after embolization and subsequent radiotherapy	yes, OCT LAR 30 mg every 28 days	- lesions operated (a,c): no persistence, no relapse - lesion not operated (b): 4 mm growth in 7 yrs at CT
**Case 2 (T.E.)**	(1) M (2) 44 (3) 48 (4) 48	44	(1) 4 (2) a: left laterocervical b: right carotid bifurcation c: right jugular foramen d: cervical	a: NA b: 6 c: NA d: NA	- echography - CT angiography (c and d lesion were detected exclusively by functional imaging)	M: 50 µg/24 h (50–340), N: 239 µg/24 (90–445)	NA	a: yes, 2015 b: no c: no d: no	no	- lesion operated (a): no persistence, no relapse - lesion not operated (b): echography, 6.5 mm (Nov 2018); MR angiography 20 mm (April 2019)
**Case 3 (T.I.)**	(1) F (2) 19 (3) 16 (4) 19	15	(1) 2 (2) a, b: left laterocervical	a: 30 b: 15	- echography - MR angiography	M: 34 µg/24 h (50–340), N: 163 µg/24 (90–445)	NA	a, b: yes, 2019	no	NA
**Case 4 (T.M.)**	(1) F (2) 36 (3) 44 (4) 49	156	(1) 3 (2) a: left laterocervical b: at 1.5 cm of the petrous canal between carotid and jugular c: right carotid glomus	a: NA b: 13 c: 16	- echography - CT angiography - MR angiography	M: 57 µg/24 h (50–340), N: 104 µg/24 (90–445)	a: NA c: paraganglioma S100+; 1 mitosis/50 HPF	a: yes, 2006 b: no c: yes, 2007	no	- lesions operated (a, c): no persistence, no relapse -lesion not operated (b): 11 mm growth in 7 yrs at CT
**Case 5 (T.R.)**	(1) M (2) 50 (3) 47 (4) 52	26	(1) 1 (2) a: left carotid bifurcation	a: 20	- echography - MR angiography - CT angiography	M: 62 µg/24 h (50–340), N: 62 µg/24 (90–445)	a: Paraganglioma S100+; Synaptophysin +; CgA +/−	a: yes, 2018	No	- lesion operated (a): no persistence, no relapse

**Table 2:** Diagnostic performance of 68Ga-DOTATOC PET-CT, 18F- FDG PET-CT and 18F-DOPA PET-CT. Abbreviations: NP = not performed; PET = positron emission tomography; CT = computed tomography; DOTATOC = 1,4,7,10-tetraazacyclododecane-1,4,7,10-tetraacetic acid tyr3- Octreotide; FDG = fluoro-2-deoxy-D-glucose; DOPA = 3,4-dihydroxyphenylalanine; SUV = standardized uptake value. * = the lesion(s) appeared later during the follow-up and no 68Ga-DOTATOC PET-CT was performed.

Patient	Lesions (Number)	^68^Ga-DOTATOC PET-CT	^18^F- FDG PET-CT	^18^F-DOPA PET-CT
**All**	13	9/9 (7 confirmed at morphological imaging and 2 not confirmed at morphological imaging) *(4 NP)*	5/6 (mean SUV max 9.2) *(7 NP)*	3/4 (confirmed at morphological imaging) *(9 NP)*
**Case 1 (T.D.)**	3	3/3	2/3	2/3
**Case 2 (T.E.)**	4	3/3 (2 not confirmed at morphological imaging) (1 NP) *	NP	NP
**Case 3 (T.I.)**	2	2/2	2/2	NP
**Case 4 (T.M.)**	3	1/1 (2 NP) *	1/1	NP
**Case 5 (T.R.)**	1	NP	NP	1/1
